# Morphological and metabolic changes in Changshan Huyou (*Citrus changshan-huyou*) following natural tetraploidization

**DOI:** 10.1186/s12870-025-06293-4

**Published:** 2025-03-08

**Authors:** Peiru Huang, Tianyu Xu, Gang Wang, Lin Zhang, Ying Yao, Min Zhang, Chi Zhang

**Affiliations:** 1 Key Laboratory of Quality and Safety Control for Subtropical Fruit and Vegetable, Ministry of Agriculture and Rural Affairs, College of Horticulture Science, Zhejiang A&F University, Hangzhou, Zhejiang 311300 People’s Republic of China; 2 Present Address: Collaborative Innovation Center for Efficient and Green Production of Agriculture in Mountainous Areas of Zhejiang Province, College of Horticulture Science, Zhejiang A&F University, Hangzhou, Zhejiang 311300 People’s Republic of China; 3 State Key Laboratory of Subtropical Silviculture, Zhejiang A&F University, Hangzhou, Zhejiang 311300 People’s Republic of China; 4 Agriculture and Rural Bureau of Changshan County, Quzhou, Zhejiang 324200 People’s Republic of China; 5 Zhejiang Agricultural Technology Extension Center, Hangzhou, Zhejiang 310020 People’s Republic of China

**Keywords:** Changshan Huyou, Tetraploid, Morphology, Anatomy, Metabolome, Transcriptome

## Abstract

**Background:**

Polyploids in citrus are generally used to improve crop varieties. Changshan Huyou (*Citrus changshan-huyou*) is a native citrus species in China that is highly adaptable and has pharmaceutical value. However, the influence in Changshan Huyou following polyploidization remains unclear. Here we evaluated the adult tetraploid scions of Changshan Huyou with contemporary diploid scions as the control in the phenotypic variations, metabolic alterations of fruits and associated transcriptomic changes.

**Result:**

The tetraploid scions had rounder and thicker leaves, larger floral organs and fruits, and satisfactory viability of pollen grains and ovules. The tetraploid fruits accumulated lower levels of soluble solids but similar levels of organic acids. Metabolic profiling of three tissues of fruits revealed that most of 2064 differentially accumulated metabolites (DAMs), including flavonoids, lignans, and coumarins, were downregulated. In contrast, the upregulated DAMs mainly included alkaloids (clausine K and 2-(1-pentenyl)quinoline), amino acids (L-asparagine and L-ornithine), and terpenoids (deacetylnomilin and evodol) in tetraploid peels, as well as, flavonoids (neohesperidin and quercetin-5-O-β-D-glucoside) and organic acids (2-methylsuccinic acid and dimethylmalonic acid) in juice sacs. The upregulated genes were associated with phenylpropanoid biosynthesis, secondary metabolite biosynthesis, and the biosynthesis of various alkaloid pathways. Pearson Correlation Analysis showed that the upregulated genes encoding *PEROXIDASE* and *CYTOCHROME P450* (*CYP450*) were closely related to the higher accumulation of amino acids and alkaloids in tetraploid peels, and up-regulated neohesperidin and quercetin glucoside were positively associated with *FERULATE-5-HYDROXYLASE* (*F5H*), *CYP450* 81Q32, *FLAVONOID 3'-MONOOXYGENASE* (*F3'H*), *4-COUMARATE–CoA LIGASE* 1 (*4CL1*), and *UDP-GLUCOSE FLAVONOID 3-O-GLUCOSYLTRANSFERASE* (*UFOG*), as well as, some transcription factors in tetraploid juice sacs.

**Conclusion:**

The tetraploid Changshan Huyou investigated here may be used in triploids breeding to produce seedless citrus, and for fruit processing on pharmaceutical purpose due to the alteration of metabolites following polyploidization.

**Supplementary Information:**

The online version contains supplementary material available at 10.1186/s12870-025-06293-4.

## Introduction

A polyploid is an organism with more than two sets of complete chromosomes, and polyploidy is ubiquitous in plants in the wild [1–4]. Polyploidy is important for ecology and evolution and is a frequently used speciation approach [5]. There are usually two types of polyploids, including allopolyploids and autopolyploids. Allopolyploids help manage evolutionary alterations and enhance species survival due to their dual effects of hybridization and polyploidy [6–8], whereas, autopolyploids usually show greater adaptability to short-term changes caused by changes in the metabolic phenotype [9] mostly subjected to whole-genome duplication [10].

Tetraploidy-mediated regulation of gene expression and epigenetic remodeling in polyploid genome can induce morphological and physiological alterations in citrus plants, such as an increase in organ size, a decrease in plant height, and diverse anatomical characteristics [11–13]. These alterations were found to increase plant growth in autotetraploid *Poncirus trifoliata,* Carrizo citrange*, Citrus wilsonii*, and *Citrus junos* ‘Ziyang’ due to their improved tolerance to environmental stress, such as drought, salinity, and heavy metals [14–19] and even biotic stress, such as Huanglongbing (HLB) infection [20], which is a fatal disease that affects various citrus species worldwide, and promising potential applications in citrus rootstocks that are adaptive to increasingly poor planting circumstances. Additionally, the essential oil composition could be dramatically modified in tetraploid *Citrus limon* induced by colchicine [21] and in tetraploid *Citrus sinensis*, suggesting that ploidy levels may contribute to the profiling of aromatic flavors in citrus [22].

Autopolyploidization may contribute to an increase in plant reproduction, which can subsequently improve distant hybridization and colonization of plants [23] and is extensively used in citrus breeding through spontaneous doubling or mutation [12, 17, 24–26]. Additionally, autotetraploid germplasm can occur spontaneously or synthetically and is crucial to triploid breeding in citrus. Doubled diploid citrus plants frequently generate pollen grains that are less fertile than the initial diploid genotypes [27, 28], however, the viability of pollen grains is adequate for the application as male parents during sexual interploid hybridization. Polyploidization can restore fertility among emerging hybrids [29]. The mechanism underlying the formation of the 2n megagametophyte contributes to optimizing sexual polyploid hybridization in citrus [30].

Changshan Huyou (*Citrus changshan-huyou,* diploid) is a native citrus species cultured for many years in local China and is characterized by vigorous growth, frost tolerance, strong adaptability, and storage resistance [31, 32]. Changshan Huyou is in a highly similarity to sour oranges (*Citrus aurantium*) formed by the hybridization of *Citrus grandis* and mandarin [33]; the juice of the mature fruit is a good source of bioactive polyphenols, vitamin C, and folic acid [31, 32]. Moreover, the dried immature fruits of Changshan Huyou, known as Quzhou Aurantii Fructus (QQAF) or *Qu zhi ke* (QZK), were recorded in the Chinese Pharmacopoeia as medicinal materials in 2015 [32, 34] due to the presence of bioactive components, such as flavonoids, volatile oils, coumarins, terpenes (especially limonoids), and steroid glycosides [31, 32, 34–37]. They also exert pharmacological effects, such as antioxidative, antibacterial, anti-inflammatory [34, 36], antitumor [38], hypoglycemic [39], and hypotensive effects, along with their ability to treat non-alcoholic fatty liver [40, 41].

In this study, a tetraploid seedling (4X) was identified from the diploid (2X) seedlings of Changshan Huyou, which had transitioned to adult phase. The morphological features, reproductivity, and fruit quality of the tetraploid Changshan Huyou remain undescribed. To get knowledge of the tetraploid plant, we investigated the morphological characteristics and metabolic profiles of fruits between the 4X scions and the 2X scions grafted with the same rootstocks, respectively, as well as the underlying changes in the transcriptome of Changshan Huyou subjected to polyploidization. Our findings showed the features of the seedlings and fruits of the 4X scions of Changshan Huyou and prediction of its superiority as the male and female parent in ploidy hybridization of triploid citrus breeding, as well as, its applications as pharmaceutical alternatives to diploid fruits in the citrus industry.

## Results

### Ploidy confirmation and genetic identification of 4X Changshan Huyou

**Fig. 1:**
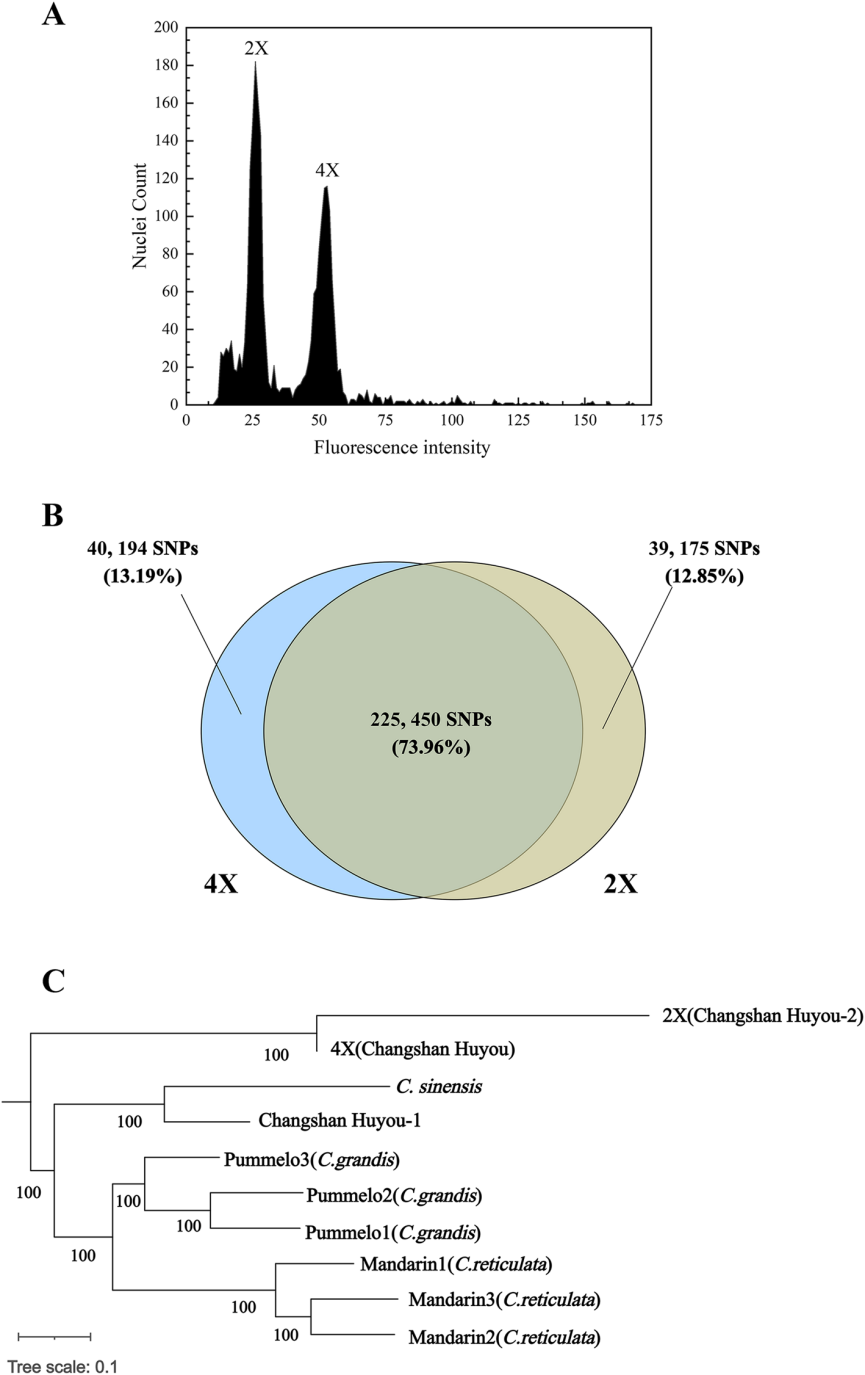
Ploidy levels and genetic constitution analysis of the tetraploid Changshan Huyou. **A **Ploidy level of the tetraploid and the diploid (control) determined by flow cytometry (FCM). **B** Venn diagram of SNP number and proportion between the diploid and the tetraploid. **C** A phylogenetic tree constructed according to the single nucleotide polymorphisms (SNPs) of the diploid (2X), the tetraploid (4X), three mandarins (*Citrus reticulata*) (NCBI GenBank ID: 44977118 for mandarin 1, 53161798 for mandarin 2, and 53161808 for mandarin 3), and three pummelos (*Citrus grandis*) (NCBI GenBank ID: 4185428 for pummelo 1, 42092598 for pummelo 2, and 44972828 for pummelo 3), Changshan Huyou-1 (NCBI GenBank ID: 1094464) and *C. sinensis* Osbeck (NCBI GenBank ID: 347609) against the Wanbaiyou (*Citrus grandis*) reference genome (http://citrus.hzau.edu.cn/download.php)

The ploidy status was checked and confirmed through flow cytometry (FCM), and the result indicated that the fluorescence intensity of the 4X cells peaked at about 50, a value that was double of that in the 2X control, suggesting a tetraploid seedling of doubled genome of 2X Changshan Huyou(Fig. 1A).

The genome of the 4X seedling and the diploid progenitor were resequenced to characterize the genetic fidelity and genomic composition of tetraploids. As the Changshan Huyou genome sequence was unavailable, we used the Wanbaiyou genome as a reference to identify single nucleotide polymorphism (SNP) in 2X and 4X. Additionally, SNPs were detected using the resequencing data of three pummelos and three mandarins against the Wanbaiyou genome, respectively. When the genomic sequences were aligned, the 2X and the 4X shared 225,450 SNPs, representing 73.96% sequence identity (Fig. 1B). Based on the phylogenetic analysis of SNPs obtained from 2X (Changshan Huyou-2), 4X (Changshan Huyou), three pummelos, three mandarins, Changshan Huyou-1 and *C. sinensis* Osbeck, respectively, the 4X was closest to the 2X, and closer to the cluster of Changshan Huyou-1 and *C. sinensis* (Fig. 1C). These findings suggested that the 4X seedling was a tetraploid that originated from natural doubling of diploid genome of Changshan Huyou.

### Morphological features of 4X leaves, flowers, and fruits

**Fig. 2:**
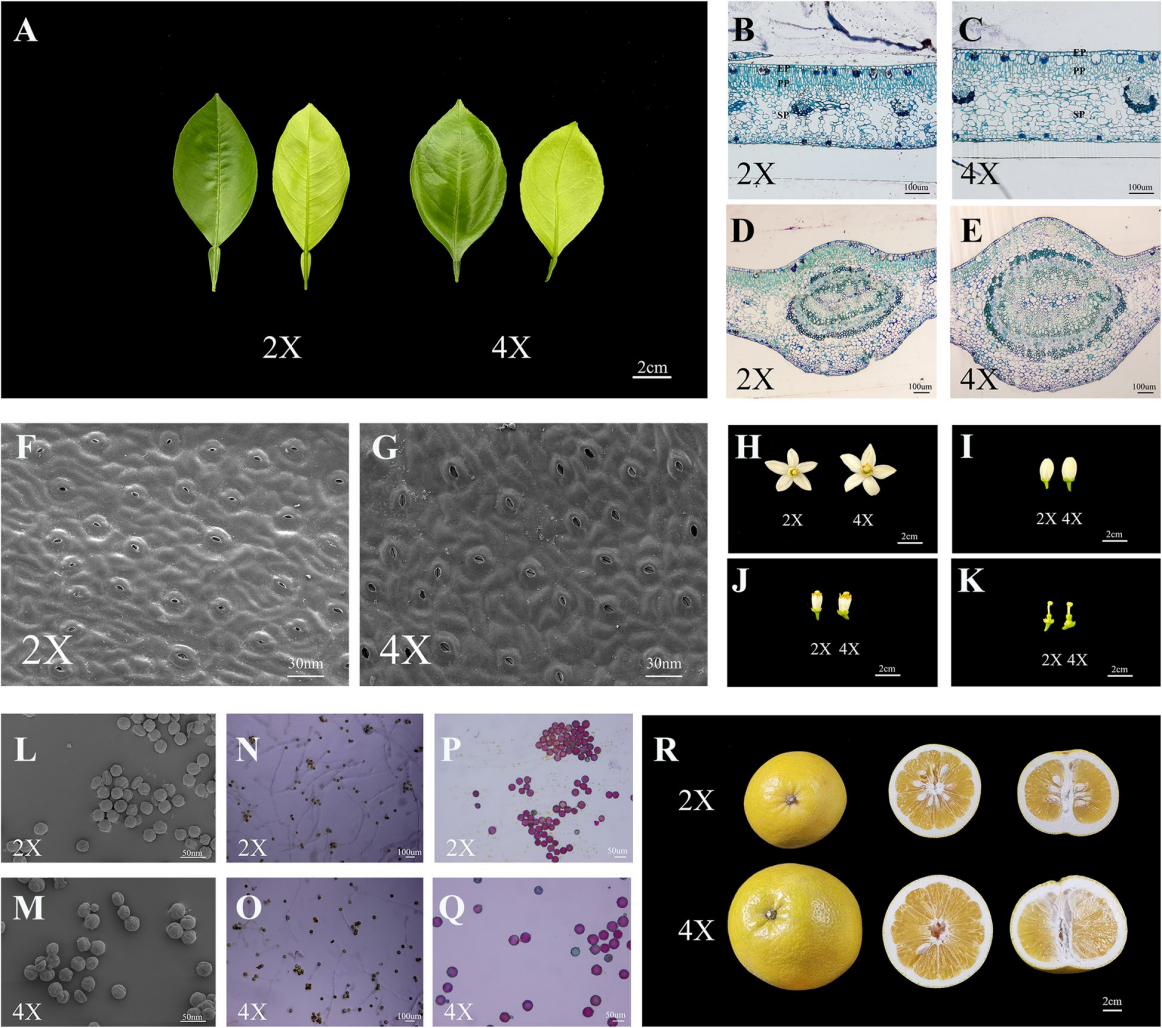
**A** Morphology of the leaves of the diploid (2X) and the tetraploid (4X) (Bar = 2 cm).The leaf was showed in both adaxial (upper) (left) and abaxial (lower) (right) surfaces. **B-E** Transversal sections of leaves (**B**-**C**) and midribs (**D-E**) of the diploid (2X) and the tetraploid (4X) (Bar = 100 μm). **F-G** Scanning electron micrographs of stoma of the diploid (2X) and the tetraploid (4X) (Bar = 30 nm). EP: Epidermis, PP: Palisade parenchyma, SP: Spongy parenchyma. **H–K** Morphology of blooming flowers (**H**), floral buds (**I**), stamens (**J**), and pistils (**K**) of the diploid (2X) and the tetraploid (4X) (Bar = 2 cm). **L, M** Scanning electron micrographs of pollen grains of the diploid (2X) and the tetraploid (4X) (Bar = 50 nm).(**N, O**) The pollen germination of the diploid (2X) and the tetraploid (4X) (Bar = 100 μm). **P**,** Q** Pollen staining activity of the diploid (2X) and the tetraploid (4X) (Bar = 50 μm). **R** Morphological characteristics of fruits in the diploid (2X) and the tetraploid (4X) (Bar = 2 cm)

Grafted with 2-year-old *Poncirus trifoliata* seedlings and grown under normal greenhouse conditions, the 4X scions presented morphological differences in their leaves, floral organs, and pollen grains compared to the 2X scions (Table 1). The 4X leaves were significantly rounder (6.47 cm ± 0.60 cm) and had a lower leaf index (1.57 ± 0.13) (Fig. 2A; Table 1). Microscopic analysis suggested distinct anatomical heterogeneities in the 4X leaves versus the 2X leaves, as determined by the thicker epidermis and larger palisade tissue cells in the 4X leaves (Fig. 2B-E; Table 1). Specifically, the 4X leaf had a thicker upper epidermis (16.07 μm ± 2.72 μm), larger palisade parenchyma (79.58 μm ± 5.49 μm) and spongy parenchyma (254. 36 μm ± 8.09 μm) (Table 1, Fig. 2B-C). The midrib diameter of the 4X leaf (1386.69 μm ± 37.28 μm) was greater than that of the 2X leaf (864.17 μm ± 17.37 μm) (Table 1, Fig. 2D-E). Additionally, the 4X leaf presented a significantly lower stomatal density (Fig. 2F-G) and significantly longer and wider guard cells than that 2X leaf (Table 1).

**Table 1:** Comparison of morphological characteristics of leaves in the diploid (2X) and the tetraploid (4X)

Strains	Leaf Blade (cm)		Shape Index of Leaf	Blade Thickness (μm)	Guard Cell (μm)		Guard Cell Density (No./mm^2^)	Epidermis (μm)		Palisade Parenchyma (μm)	Spongy Parenchyma (µm)	Midribs (µm)
	Length	Width			Length	Width		Upper	Lower			
2X	11.31 ± 1.09	6.01 ± 0.69	1.9 ± 0.21	344.15 ± 8.77	21.25 ± 2.11	18.27 ± 1.07	432.45 ± 36.32	11.04 ± 2.01	9.29 ± 1.86	70.59 ± 3.96	241.10 ± 8.47	864.17 ± 17.37
4X	10.13 ± 0.75	6.47 ± 0.60	1.57 ± 0.13	376.70 ± 7.80	25.91 ± 2.44	22.49 ± 2.16	371.14 ± 32.94	16.07 ± 2.72	9.91 ± 1.71	79.58 ± 5.49	254.36 ± 8.09	1386.69 ± 37.28
*t*-test	NS	**	**	**	**	**	**	**	NS	**	**	**

Analysis of variance (ANOVA) and student’s t-test were used to detect the difference between genotypes, indicate by * (*P* < 0.05) and ** (*P* < 0.01) as significance, and NS as no significance, respectively. The data are presented as mean ± standard deviation (n = 18). 2X: diploid, 4X: tetraploid

In terms of the floral organ number, no significant difference between the 4X scions and the 2X scions, however, the size of organs, such as petals, stamens, and ovaries, was larger in the 4X scions (Table 2, Fig. 2H-K). The length and width of petals were significantly greater in 4X than those in 2X (Table 2, Fig. 2H), resulting in longer flower buds in the 4X (Table 1, Fig. 2I). The stamens were considerably longer in 4X than the stamens in 2X (Table 2, Fig. 2J). The pistil had no significant difference between the 4X and the 2X, whereas the vertical and transverse diameters of the 4X ovules were greater (Table 2, Fig. 2K). The 4X had larger pollen grains (Fig. 2M, Table 2) and a little lower viability in the staining and germination of pollen grains (Fig. 2N-Q, Table 2).

**Table 2:** Comparison of morphological characteristics of flowers and pollen in the diploid (2X) and the tetraploid (4X)

Strains	Flower Bud (cm)		No. of Petal	Petal (cm)		Length of stamen (cm)	Length of Pistil (cm)	Ovary (cm)		No. of Stamens	Pollen Grain (μm)		Shape Index of Pollen Grain	Pollen Stability Rate (%)	Pollen Germination Rate (%)
	Length	Width		Length	Width			Diameter	Height		Length	Width			
2X	2.00 ± 0.21	1.04 ± 0.12	5.00 ± 0.00	1.98 ± 0.15	0.84 ± 0.12	1.24 ± 0.14	1.28 ± 0.26	0.43 ± 0.04	0.56 ± 0.05	24.08 ± 2.22	25.68 ± 1.55	23.85 ± 1.76	1.08 ± 0.09	86.4 ± 9.42	44.73 ± 7.77
4X	2.11 ± 0.22	1.13 ± 0.07	5.00 ± 0.47	2.28 ± 0.14	1.04 ± 0.13	1.33 ± 0.13	1.41 ± 0.16	0.49 ± 0.07	0.61 ± 0.10	24.20 ± 1.92	29.16 ± 1.99	26.57 ± 2.84	1.11 ± 0.10	85.03 ± 12.43	33.43 ± 6.40
*t*-test	*	NS	NS	NS	NS	*	NS	*	*	NS	**	**	NS	NS	**

Analysis of variance (ANOVA) and student’s t-test were used to detect the difference between genotypes, indicate by * (*P* < 0.05) and ** (*P* < 0.01) as significance, and NS as no significance, respectively. The data are presented as mean ± standard deviation (n = 18). 2X: diploid, 4X: tetraploid

Fruits from the 4X scions were much larger than those from 2X scions (Table 3, Fig. 2R). The edible rate of fruits from the 4X was similar with that from the 2X; however, the average fruit weight of fruits from the 4X was double that from 2X because of the larger diameter and height of fruits from the 4X. No significant difference was recorded in the TA (titratable acidity) of juice sac; however, the SSC (soluble solids content) of juice sac was significantly lower in the fruits from the 4X scions than that from the 2X scions. Particularly, fewer developed seeds and greater undeveloped seeds were derived from the 4X fruits (Table 3).

**Table 3:** Comparison of the morphological characteristics of fruits in the diploid (2X) and the tetraploid (4X)

Strains	Fruit Weight (g)	Fruit(cm)		Shape Index of Fruit	Juice Sac Weight (g)	Edible Rate (%)	No. of Segments	No. of Developed Seeds/Fruit	No. of Undeveloped Seeds/Fruit	Rate of developed Seeds/Fruit	SSC of Juice Sac (%)	TA of Juice Sac (%)
		Diameter	Height									
2X	318.90 ± 31.36	9.70 ± 0.42	9.17 ± 1.01	0.95 ± 0.10	227.98 ± 1.72	72.15 ± 5.23	10.00 ± 0.82	21.17 ± 12.84	2.17 ± 1.47	0.84 ± 0.12	10.77 ± 0.15	1.41 ± 0.15
4X	676.46 ± 70.65	13.05 ± 0.48	11.32 ± 0.69	0.87 ± 0.56	488.07 ± 1.60	71.49 ± 3.47	9.55 ± 1.21	6.50 ± 4.60	8.86 ± 8.16	0.39 ± 0.19	8.91 ± 0.09	1.56 ± 0.16
*t*-test	**	**	**	**	**	NS	*	*	*	**	**	NS

Analysis of variance (ANOVA) and student’s t-test were used to detect the difference between genotypes, indicate by * (*P* < 0.05) and ** (*P* < 0.01) as significance, and NS as no significance, respectively. The data are presented as mean ± standard deviation (*n* = 18). 2X: diploid, 4X: tetraploid

### Differentially accumulated metabolites (DAMs) between tetraploid and diploid fruits

**Fig. 3:**
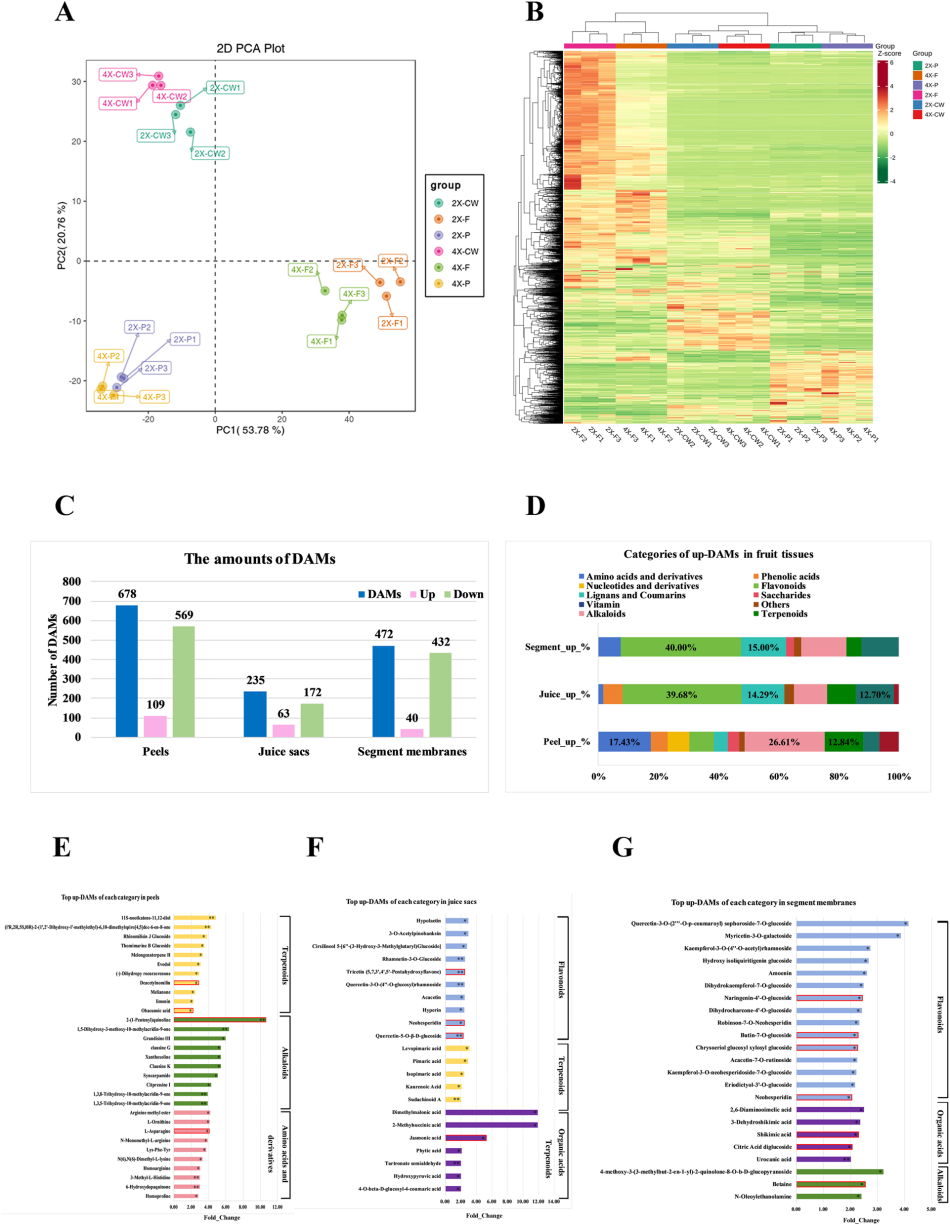
Comparative metabolic profiling in peels, juice sacs and segment membranes between tetraploid and diploid. **A **Principal component analysis (PCA) of all samples from three tissues of fruits. **B** Hierarchical cluster analysis (HCA) of all metabolites identified in three tissues of fruits. **C** The amounts of differentially accumulated metabolites (DAMs), up-regulated DAMs and down-regulated DAMs. **D** Categories of up-regulated DAMs in fruit tissues. **E–G** Representatives of up-regulated DAMs in peels, juice sacs, and segment membranes, respectively. 4X-P, tetraploid juice sacs; 2X-P, diploid juice sacs; 4X-F, tetraploid peels; 2X-F, diploid peels; 4X-CW, tetraploid segment membranes; 2X-CW, diploid segment membranes. An asterisk (*) represents a significance with *p*-value at the 0.05 level, and two asterisks (**) represent a significance with *p*-value at the 0.01 level

Fruits are crucial products of Changshan Huyou cultivation. Thus, we analyzed whether the 4X fruits presented an improved metabolic profile than the 2X fruits. Three fruits were collected from one independent tree for one duplicate, and the triplicates were obtained from three independent tress of 2X and 4X scions, respectively. Comprehensive metabolites were investigated in the peels, juice sacs, and segment membranes of fruits derived from the 4X scions and the 2X scions, respectively, harvested at 210 days after flowering (DAF) by broadly targeted metabolomics using a UPLC-MS/MS system. The differences in the metabolic profiles of three fruit tissues of different ploidy level were assessed by PCA (Principal Component Analysis), and 53.78% of the variables among the three tissues could be explained by the first component, whereas 20.76% could be explained by the second component (Fig. 3A). The PCA in Fig. 3A indicated that the metabolites from 4X juice sacs (4X-P) and 2X juice sacs (2X-P) were in a high similarity, and in contrary, the metabolites were relatively different in comparison between 4X peels (4X-F) and 2X peels (2X-F), so did in comparison between 4X segment membranes (4X-CW) and 2X segment membranes (2X-CW), suggesting the tetraploidy modified the metabolites in a small scale, and more changes were involved in peels and segment membranes in our tetraploid Changshan Huyou. Besides, the PCA also indicated that the metabolites were quite different between tissues, indicating a significant metabolic difference between tissues and distinct functions of individual tissue.

In total, 2064 metabolites grouped into 13 primary classes, such as amino acids or their derivatives, nucleotides or their derivatives, phenolic acids, flavonoids, etc., were identified in three parts of the fruits (Additional file 1). The total contents of flavonoids and organic acids did not change significantly in 4X juice sacs (Additional file 2A), whereas the contents of flavonoids, lignans, coumarins, and vitamins decreased significantly in 4X peels (Additional file 2B), and the contents of quinones, saccharides, alkaloids, and lipids decreased significantly in the 4X segment membranes (Additional file 2C).

In total, 678 (32.8% of total metabolites of 2064), 235 (11.4%), and 472 (22.9%) DAMs were screened from the peels (4X-F_vs_2X-F), juice sacs (4X-P_vs_2X-P), and segment membranes (4X-CW_vs_2X-CW), respectively (Additional file 3, Fig. 3B). Different patterns of metabolite accumulation were found in different tissues. However, the number of upregulated DAMs in 4X tissues (up-DAMs) was considerably lower than that of downregulated DAMs in 4X tissues (down-DAMs) (Fig. 3C, Additional file 4). In the three tissues, the major categories of down-DAMs were flavonoids, lignans, and coumarins; however, the dominant categories of up-DAMs were different between the tissues. In peels, the up-DAMs mainly consisted of alkaloids, terpenoids, and amino acids or their derivatives (Fig. 3D), represented by 2-quinoline (Wagp010286, with a 10.2-fold change), L-asparagine (mws0001, 4.1), and deacetylnomilin (Cmpp004617, 2.84) (Fig. 3E). In the juice sacs, flavonoids were the top category in the up-DAMs, followed by terpenoids and organic acids (Fig. 3D), and the representative flavonoids included neohesperidin (pme0001, 2.45), hyperin (MWSHY0113, 2.46), and rhamnetin-3-O-glucoside (Lmjp002906, 2.53) (Fig. 3F). In segment membranes, besides flavonoids, alkaloids and organic acids were the dominant categories in up-DAMs (Fig. 3D), and the representatives included flavonoids such as naringenin-4'-O-glucoside (HJN087, 2.44), butin-7-O-glucoside (HJN090, 2.29), and neohesperidin (pme0001, 2.04), alkaloids such as betaine (MWSmce548, 2.55) and 4-methoxy-3-(3-methylbut-2-en-1-yl)–2-quinolone-8-O-b-D-glucopyranoside (Lwhp011001, 3.22), and organic acids such as shikimic acid (mws0154, 2.30) and citric acid diglucoside (WaYn000716, 2.06) (Fig. 3G).

### Differentially expressed genes (DEGs) between fruits from 4X scions and 2X scions

**Fig. 4:**
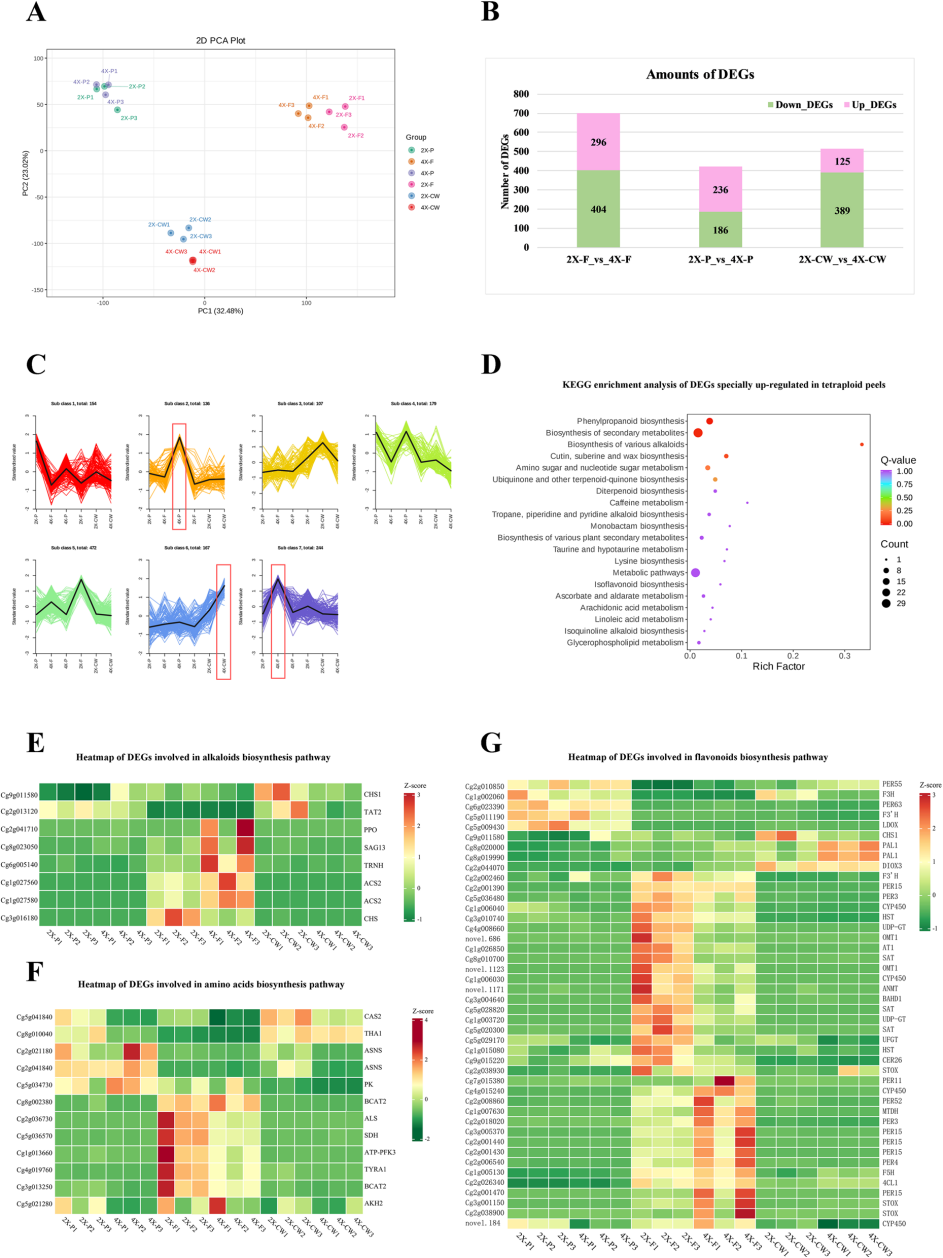
Comparative tramscriptome profiling in peels, juice sacs and segment membranes between tetraploid and diploid. **A** Principal component analysis (PCA) of all samples from three tissues of fruits. **B** The amounts of differentially accumulated metabolites (DAMs), up-regulated DAMs and down-regulated DAMs. **C** K-means analysis of all differentially expressed genes (DEGs) identified from three tissues of fruits of tetraploids and diploids. **D** KEGG enrichment analysis of DEGs specially up-regulated in tetraploid peels. **E–G** Heatmap of DEGs involved in alkaloids, amino acids, and flavonoids biosynthesis pathways, respectively. 4X-P, tetraploid juice sacs; 2X-P, diploid juice sacs; 4X-F, tetraploid peels; 2X-F, diploid peels; 4X-CW, tetraploid segment membranes; 2X-CW, diploid segment membranes

To assess the underlying alterations driven by ploidy in fruits, the global transcriptomic profiles were analyzed using the corresponding tissues of tetraploid and diploid fruits. We acquired 125.18 Gb of clean data from 18 cDNA libraries of quality-filtered sequence data, and > 89.22% of the bases had a quality score of ≥ Q30 (Additional file 5). PCA of gene expression via FPKM showed that three tissues of fruits were clustered separately, and the first two components explained 55.50% of the variance (Fig. 4A). Besides the separate clustering of the three tissues, the PCA in Fig. 4A indicated that the gene expression was similar in fruit juice sacs between 4 and 2X, and relatively more different in fruit peel and segment membrane between 4 and 2X, respectively. In total, 700, 422, and 514 DEGs were filtered from pairwise comparisons of 2X-F_vs_4X-F, 2X-P_vs_4X-P, and 2X-CW_vs_4X-CW, respectively (Additional file 6). Similar to DAMs, there were more downregulated DEGs (down-DEGs) in the 4X tissues than upregulated DEGs (up-DEGs) in the 4X tissues (Fig. 4B, 2X-F_vs_4X-F, 2X-CW_vs_4X-CW), however, the up-DEGs exceed over the down-DEGs in 4X juice sacs (Fig. 4B, 2X-P_vs_4X-P). Gene Ontology (GO) analysis revealed that DEGs from different fruit tissues were related to cellular processing and biological metabolic processes, as well as, to the molecular functions of catalytic activity and binding (Additional file 7). According to the Kyoto Encyclopedia of Genes and Genomes (KEGG) analysis, the most enriched pathway was associated with the biosynthesis of secondary metabolites in all three tissues (Additional file 8). The results of K-means analysis revealed that the DEGs were clustered into seven groups, and the DEGs upregulated only in tetraploid juice sacs, segment membranes, and peels were grouped into subclasses 2, 6, and 7, respectively (Additional file 9, Fig. 4C). The DEGs that were upregulated only in the 4X-F (subclass 7) were enriched in the biosynthesis of secondary metabolites; phenylpropanoid biosynthesis; the biosynthesis of various alkaloids; cutin, suberin, and wax; amino sugar and nucleotide sugar metabolism; ubiquinone and other terpenoid-quinone biosynthesis (Additional file 10, Fig. 4D). However, the number of upregulated DEGs was lower in KEGG enrichment analysis of 4X-P (Additional file 11, 12A) and 4X-CW (Additional file 12B, 13) groups than that of 4X-F group.

Apart from *TYROSINE AMINOTRANSFERASE 2* (*TAT2*, Cg2g013120) and *CHALCONE SYNTHASE* (*CHS1*, Cg9g011580; *CHS*, Cg3g016180), DEGs related to alkaloid biosynthesis (ko00996) were mainly expressed and upregulated in 4X-F (Fig. 4E). Amino acid biosynthesis (ko01230)-related DEGs showed two gene expression patterns that were expressed mainly in peels and whole fruits (Fig. 4F). The former, which included *BRANCHED-CHAIN AMINO ACID AMINOTRANSFERASE* (*BCAT*, Cg3g013250 and Cg8g002380), *AROGENATE DEHYDROGENASE 1* (*TYRA*, Cg4g019760), and *ACETYLACTATE SYNTHASE I* (*ALS*, Cg2g036730), were expressed at relatively high levels in 2X-F. The latter included *CYSTEINE SYNTHASE 2* (*CAS2*, Cg5g041840) and *THREONINE ALDOLASE* (*THA1*, Cg8g010040), whose levels were relatively higher in 2X-P and 2X-CW; whereas the levels of *ASPARAGINE SYNTHASE* (*ASNS*, Cg2g021180 and Cg2g041840) and *PYRUVATE KINASE ISOZYME A* (*PK*, Cg5g034730) were higher in 4X-P. Flavonoid biosynthesis was associated with the greatest number of DEGs expressed in peels (2X-F and 4X-F) and whole fruits, among which *CYTOCHROME P450* (*CYP450*, Cg4g015240), *FERULATE-5-HYDROXYLASE* (*F5H*, Cg1g005130), *FLAVONOID 3'-HYDROXYLASE* (*F3’H*, Cg2g002460), and *4-COUMARATE–CoA LIGASE 1* (*4CL1*, Cg2g026340), were specifically upregulated in 4X-F (Fig. 4G).

### Correlations reveal significant interactions between up-DAMs and DEGs enriched in 4X fruits

**Fig. 5:**
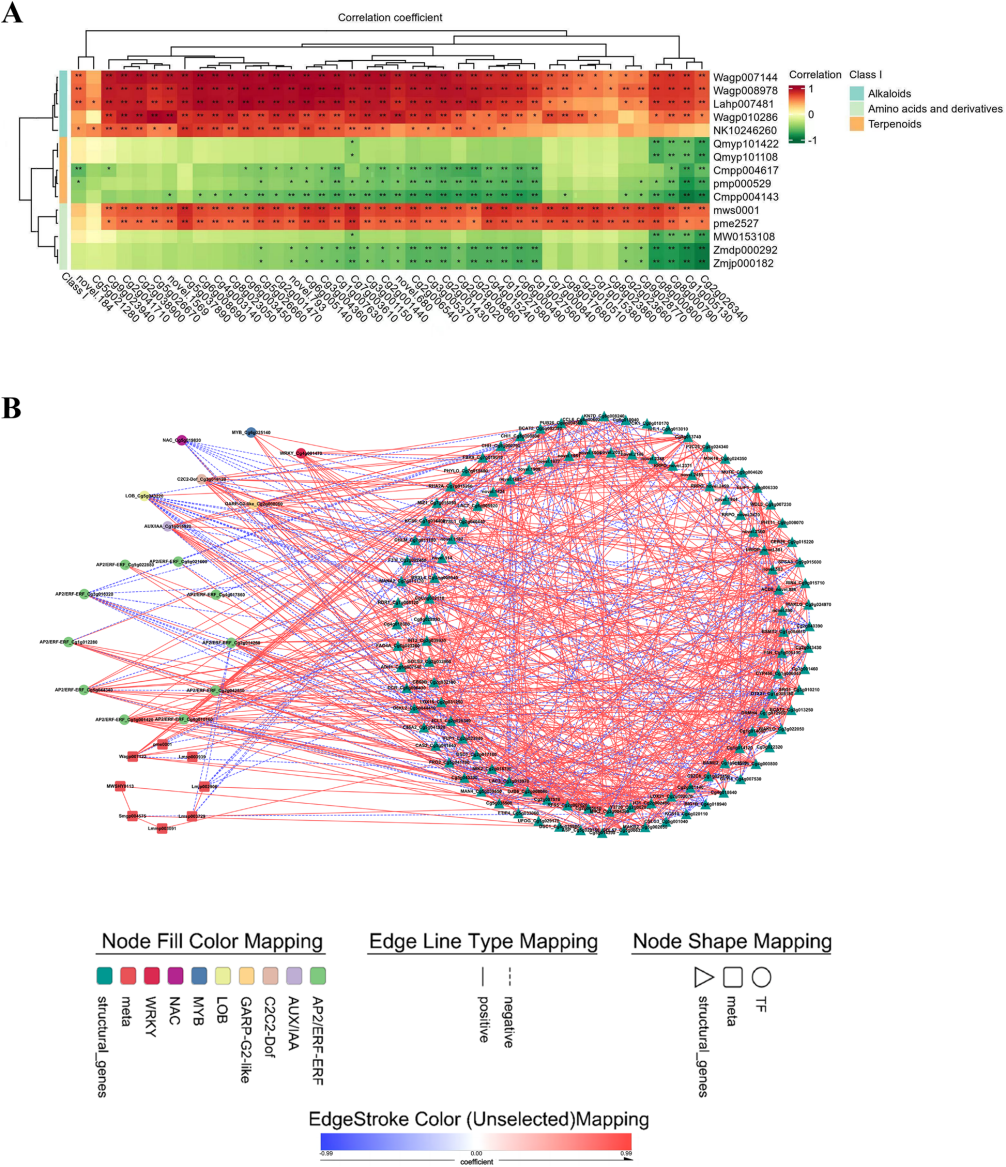
Correlation analysis of up-DAMs and DEGs related to amino acids, terpenoids, alkaloids and flavonoids. **A** Up-regulated amino acids, terpenoids and alkaloids were correlated with the up-regulated DEGs in tetraploid peels. An asterisk (*) represents a significance with *p*-value at the 0.05 level, and two asterisks (**) represent a significance with *p*-value at the 0.01 level. **B** The up-regulated flavonoids were correlated with DEGs involved in pathway of flavonoids biosynthesis in juice sacs. Lmjp002906: Rhamnetin-3-O-Glucoside; Lmmp003091: Quercetin-3-O-(4''-O-glucosyl)rhamnoside; Lmsp003729: Myricetin-3-O-rutinoside; Lmsp003939: Isorhamnetin-3-O-galactoide-7-O-rhamnoside; MWSHY0113: Quercetin-3-O-galactoside (Hyperin); pme0001: Hesperetin-7-O-neohesperidoside(Neohesperidin); Smgp004575: Quercetin-5-O-β-D-glucoside; Wagp007122: 5,4'-Dihydroxy-6,7,8,3'-tetramethoxyflavone

To determine the relationships between metabolic alterations and changes in the transcriptome, correlations were analyzed between up-DAMs and several DEG groups by Pearson’s correlation analysis with screening criteria of |PCC|> 0.85 and *P* < 0.05 (Additional file 14−16). In total, 43 up-DEGs, specifically in 4X-F, were subjected to the top five up-DAMs of each category in the comparison of 4X-F_vs_2X-F (Fig. 5A, Additional file 14). In general, 18 of the 31 upregulated DEGs encoded oxidases among the correlated genes in peels (Additional file 14). Alkaloids were positively correlated with seven *PEROXIDASE*s (Cg2g001430, Cg2g001440, Cg2g001470, Cg3g005370, Cg2g018020, Cg2g006540, and Cg2g008860); however, only two genes were negatively correlated with terpenoids. Two amino acids, L-asparagine and L-ornithine, were positively co-expressed with oxidase genes, such as *CYP450* (Cg7g003610 and Cg4g015240) and *TROPINONE REDUCTASE* (Cg6g005140).

In the juice sacs, the upregulated flavonoids, including neohesperidin (pme0001), Quercetin-5-O-β-D-glucoside (Smgp004575), and 5,4'-dihydroxy-6,7,8,3'-tetramethoxyflavone (Wagp007122), were positively correlated with upregulated DEGs, such as *F5H* (Cg1g005130), *CYP450* 81Q32 (Cg1g006040), *F3’H* (Cg2g002460), *4CL1* (Cg2g026340), *UDP-GLUCOSE FLAVONOID 3-O-GLUCOSYLTRANSFERASE* (*UFOG*, Cg5g029170), and *PROTEIN ECERIFERUM 26* (*CER26*, Cg9g015220), which are involved in phenylpropanoid biosynthesis (ko00940), flavonoid biosynthesis (ko00941), anthocyanin biosynthesis (ko00942), isoflavonoid biosynthesis (ko00943), and flavone and flavonol biosynthesis (ko00944) pathways. These flavonoids were closely correlated with 19 transcription factors (TFs), such as *ETHYLENE-RESPONSIVE TRANSCRIPTION FACTORS* (*ERF014*, Cg3g016320), *NAC DOMAIN-CONTAINING PROTEIN 62* (*NAC62*, Cg5g019820), and *MYB16* (Cg6g025140) (Fig. 5B, Additional file 15).

## Discussion

Polyploidization in *Citrus* and its related genera usually leads to genotypes indicated by thick and round leaves, large guard cells, pollen grains, and flowers, along with poor fruit features such as thick and rough rinds and high organic acid contents relative to those of diploid plants [13, 42, 43]. In this study, the 4X scions of Changshan Huyou had the representative morphological features (Figs. 2, 3 and 4, Tables 1 and 2) and fruit qualities (Table 3) of tetraploid *Citrus* or related plants, along with the organic acid content of juice sacs, which is different from other reported tetraploid fruits in citrus [12, 13]. However, the tetraploid in this study had desirable traits for citrus breeding, such as adequate validity of the pollen grains remaining at 85.03% of staining viability and a germination rate of 33.43%, which is competent in hybridization (Table 2), as well as, ovule viability, as indicated by some developed seeds per fruit under free pollination (Table 3). Thus, the tetraploid Changshan Huyou may be developed into a new variety or can serve as an excellent male and female parent in reciprocal crosses with other diploid citrus or related genera to produce triploid hybrids with seedless characteristics, which is the typical feature of emerging citrus species [12, 44] acquired by sexual hybridization with tetraploid parents (2x × 4x, 4x × 2x, or 4x × 4x) [45] to yield genetic modifications in pharmaceutical and volatile compounds [26].

The ploidy level is strongly associated with tolerance to biotic/abiotic stress factors in citrus. The use of polyploids, especially homologous tetraploid rootstocks in citrus and related genera, increases resistance to salinity [16, 25, 46, 47], heavy metal toxicity [48, 49], and drought [50], especially in greenhouse production. These reports indicated that tetraploid rootstocks with thicker and greener leaves, lower stomatal density, larger stomata, and lower respiration rates mostly showed greater salt tolerance because of lower chloride ions (Cl^−^) accumulation in leaves and delayed damage to *Citrus macrophylla* [46], *Poncirus trifoliata* [16, 51], Carrizo citrange, and Cleopatra mandarin [16] plants. The tetraploid rootstocks of *Poncirus trifoliata*, *Citrus limonia* Osbeck, and *Citrus reshni* can sequester higher levels of chromium (Cr) into roots, accompanied by a decrease in leaf transfer rate, thus protecting the photosynthetic apparatus and green pigments from oxidative injuries [49]. Additionally, the tetraploid Swingle citrumelo (*Citrus paradisi* × *Poncirus trifoliata*) rootstock confers better resistance to HLB than its respective diploid progenitors because of fewer symptoms of HLB, limited oxidative stress, and less secondary root degradation [20]. Besides having higher metabolic levels, citrus tetraploids have higher levels of expression of stress-related genes that contribute to stress resistance [17, 24]. The tetraploids investigated in this study had good yields of fruits and seeds, of which most developed seeds can develop into tetraploid seedlings due to polyembryony, which encouraged the rootstock application of the tetraploid nucellar seedlings and may contribute to resistance of the biotic/abiotic stress in citrus production.

Alterations in metabolites following tetraploidization can influence yield, as well as, the constitution of functional metabolites in seedlings and nutrients in fruits. Polyploidization can affect the constitution of volatile organic compounds (VOCs) in Volkamer lemon (*Citrus limonia*) leaves [52], increase the content of terpenoids [21], such as limonene and cyclic monoterpenes, and improve the antioxidant activity of essential oils in *Citrus limon* [21] with modified compositions of essential oils [22]. Besides influencing seedlings, autotetraploidization can also influence metabolism in the tetraploid fruits of Ponkan mandarin (*Citrus reticulata*) [13]. Citrus fruits are rich in primary metabolites, such as sugars, organic acids, amino acids, sugar alcohols, and fatty acids, as well as, different secondary metabolites, including carotenoids, limonoids, and flavonoids. These metabolites can be used to measure the quality of citrus fruits, which are key human dietary nutrients [13, 53–55]. Consequently, evaluating the features of autotetraploid fruits is important for their use in citrus breeding. Flavonoids and carotenoids may accumulate at lower levels in tetraploid Ponkan fruits [13]; however, in a study, the tetraploid Satsuma mandarin had a relatively high content of carotenoids in the flavedo [12], which may be due to the differences in their determinate samples. In this study, the dominant categories of DAMs, especially up-DAMs (Fig. 3E-G), were significantly different between fruit tissues. In peels, alkaloids, terpenoids, and amino acids and their derivatives were positively affected in tetraploid peels (4X-F), while flavonoids and coumarins were negatively affected. Citrus peel extracts, including coumarins, flavonoids, alkaloids, and terpenes, display antibacterial and anti-inflammatory activities, whereas, amino acids exhibit pharmacological activities [53–55]. For example, L-asparagine, a key amino acid associated with the citrus green disease HLB [20], is specifically upregulated in tetraploid peels (4X-F) in this study. A tetraploid rootstock of CH (a somatic hybrid of Changsha mandarin + Benton citrange) can increase the accumulation of sugars, flavonoids, and some specific amino acids (especially asparagine), increasing the resistance or tolerance of CH to HLB [56, 57]. Additionally, L-asparagine is a promising candidate in the fields of medicine and food for reducing the production of acrylamide, which is likely carcinogenic and neurotoxic to humans [58]. Additionally, citrus flavonoids, which mainly include flavanones, flavones, and flavonols, are extremely important for human health. Neohesperidin (pme0001) is a flavonoid glycoside detected in citrus fruits; it acts as an antioxidant, greatly suppressing angiotensin II-mediated vascular remodeling and hypertension in vitro and in vivo [32]. In this study, tetraploid juice sacs (4X-P) accumulated flavonoids such as neohesperdin at relatively high levels and simultaneously presented higher expression of *F3'H* and several TFs, such as WRK40 and MYB16. Moreover, polymethoxyflavones (PMFs) are a class of abundant specialized metabolites with remarkable anticancer properties in citrus flavonoids [59]. *FLAVONOID O-METHYLTRANSFERASE*s (*OMT*s), *FLAVONOID HYDROXYLASE*s, and *FLAVONE O-DEMETHYLASE*s influence the accumulation of citrus PMFs [60–62]. The tetraploid juice sacs (4X-P) in this study showed higher accumulation of 5,4'-dihydroxy-6,7,8,3'-tetramethoxyflavone (HHWagp007122), and the abundance of the *F5H*, *CYP450*, *F3’H*, and *4CL1* were exactly increased. Overall, higher accumulation of special flavonoids were correlated to the overexpression of DEGs involved in the pathway of flavonoid biosynthesis, suggesting the enhanced biosynthesis of flavonoids in the specific tissues of tetraploid fruits and an increasing pharmaceutical applications of the tetraploid fruits due to the metabolic changes in polyploidization of Changshan Huyou.

## Conclusions

To summarize, we discovered a tetraploid Changshan Huyou and compared the morphology, physiology, metabolites, and underlying gene expression between the adult 4X scions and the contemporary 2X scions. We found that the shape, stoma, and anatomical characteristics of the 4X leaves changed, the 4X pollen grains had satisfactory viability, and the 4X fruits included certain developed seeds. Our results also showed that flavonoids, lignans, and coumarins were mainly changed in three tissues of tetraploid fruits; however, amino acids and alkaloids were the major groups enriched in upregulated DAMs in tetraploid peels. Pathway analysis indicated that genes upregulated following tetraploidization were related to phenylpropanoid biosynthesis, secondary metabolite biosynthesis, and various alkaloid biosynthesis pathways. Among them, the genes encoding *PEROXIDASE* and *CYP450* are closely related to the increased accumulation of alkaloids and amino acids in peels, and the accumulation of certain flavonoids, such as neohesperidin, quercetin glucoside, and tetramethoxyflavone, is positively related to a set of pathway genes and several TFs in tetraploid juice sacs. These findings indicated the tetraploid Changshan Huyou can be used in triploid citrus breeding for seedless varieties and pharmaceutical applications in fruit processing, demonstrating the influence on metabolites following polyploidization in Changshan Huyou.

## Materials and methods

### Plant materials

One spontaneous tetraploid (4X) genotype was originally selected among 120 seedlings grown from seeds collected from fruits harvested from a mature tree of Changshan Huyou (diploid, 2X) grown from seed in Zhejiang A&F University in 2013. Following the ploidy identification, the 4X 1-year-old scion was top-grafted with 2X 5-year-old *Poncirus trifoliata* by cut-grafting in 2014. Grown in greenhouse for 5 years, the 4X scion had successfully transitioned to adult phase. Subsequently, two grafting combination were performed to produce combined trees for comparative test, that is 4X buds and 2X buds from the adult branches grafted with 2X 2-year-old *Poncirus trifoliata*, respectively. Ten combined trees of each combination were grown in greenhouse under uniformed management of irrigation and fertilization. After 3 years vegetative growth, both the 4X scions and the 2X scions flowered. Three combined trees were selected randomly from each combination and three fruits from each combined tree were harvested for the comparative analysis on fruit appearance and quality, transcriptome and metabolome.

### Ploidy analysis

Ploidy status of seedlings were checked and confirmed by flow cytometry (Partec®, Münster, Germany) according to the methodology described by Aleza et al. [18]. The sample consist of a 0.5 cm^2^ leaf collected from the tetraploid Changshan Huyou and a similar leaf piece taken from a diploid control plant. The mixture of leaf sample were chopped in the presence of a nuclei isolation solution (High Resolution DNA Kit Type P, solution A; Partec®, Münster, Germany). Nuclei were filtered through a 30-μm nylon filter and stained with DAPI solution (4–6-diamine-2-phenylindol; High Resolution DNA Kit Type P, solution B; Partec®, Münster, Germany). Following a 5-min incubation, stained samples were injected in a Ploidy Analyzer (Partec®, PA) flow cytometer. The results were plotted on a graph using the Origin 2021 software.

### Genetic detection through single nucleotide polymorphisms (SNPs) analysis

Genomic DNA from the tetraploid (4X) and the diploid (2X) was extracted by CTAB (Cetyltrimethylammonium Bromide) method [63], respectively, and genome resequencing was performed at the Beijing Genomics Institute (BGI) (Shenzhen, China). The clean reads obtained were mapped against the Wanbaiyou (*Citrus grandis*) genome HWB.v1.0 (http://citrus.hzau.edu.cn/download.php) to identify SNPs in diploids and tetraploids. Subsequently, the genome resequencing data of three mandarins (*C. reticulata*) (NCBI GenBank ID: 44977118 for mandarin 1 *Citius mangshanensis*, 53161798 for mandarin 2 mandarin isolate UCSKl haplotype 2, and 53161808 for mandarin 3 mandarin isolate UCSKl haplotype 1) and three pummelos (*C. grandis*) (NCBI GenBank ID: 4185428 for pummelo 1 ‘Huazhouyou-tommentosa’, 42092598 for pummelo 2, and 44972828 for pummelo 3 ‘Xipi Majia’), Changshan Huyou-1 isolate 01–14 (NCBI GenBank ID: 1094464) and *C. sinensis* Osbeck HZAU DHSO 2021 (NCBI GenBank ID: 347609) were downloaded from citrus annotation project database [64] and used for SNPs identification through alignment against the same reference genome of Wanbaiyou. The SNPs were detected using MUMmer-4.0.0rc1 alignment software (version). Each sample was globally aligned with the reference genome HWB.v1.0, and initially detected the potential SNP sites; extract 100 bp sequence on each side of the reference sequence SNP site, and then compare the extracted sequence and assembly results using BLAT software to verify the SNP sites. If the length of the alignment is less than 101 bp, the SNP considered unreliable will be removed; if the alignment is repeated, the SNP considered a repeat region will also be removed; finally, the repeat region of the reference sequence is predicted by BLAST, TRF, Repeatmask software, and the SNP located in the repeat region will be filtered. Finally, SNP obtained from our resequencing and the above SNP results were integrated into ‘phy’ format files using a perl script for iqtree to build the phylogenetic tree, and the model selection was default. Loci of the common SNP for both diploid (2X) and tetraploid (4X) Changshan Huyou were counted by perl scripts and plotted by the R package VennDiagram. Next, a phylogenetic tree based on those SNPs acquired from eight genotypes was constructed using the online tool iTOL v6 (Interactive Tree Of Life, http://itol.embl.de/).

### Morphological analyses

Tetraploids and diploids grown in the identical environments were evaluated using morphological indicators, including the leaf width and length, the floral organ (floral bud, petal, pistil, ovary, petal and stamen) size and number, and the fruit weight and fruit size. Three replicates were set for each measurement.

### Scanning electron microscope (SEM) observation

Stoma SEM observations were based on three fully expanded leaves per plant and three plants per genotype (9 replicates). Slides were prepared for the analysis of stomatal size and the number of stomata per unit of leaf surface area (stomatal density) according to Oustric et al. [65]. Mature leaves were cut into pieces of 0.5 mm^2^ and fixed in the 2.5% glutaraldehyde solution, then followed gradient ethanol dehydration and drying, the leaf piece was sprayed metal and observed using SEM (SU-8010; Hitachi, Tokyo, Japan) [66]. Pollen SEM observations were performed based on three anthers collected from each plant and three plant per genotype (9 replicates) and followed description by Lora et al. [67]. Fresh pollen were released and stuck on the slide. Following the metal spray, the pollen appearance were observed using SEM.

### Longitudinal resin-embedded leaf cross-sections

Mature leaves were prepared for longitudinal resin-embedded leaf cross-sections and prepared following the described by Jiang et al. [15]. Leaves were fixed in a 2.5% glutaraldehyde solution at 4 °C overnight, followed by gradient ethanol dehydration and Spurr resin embedding. Later, 0.05% toluidine blue O (CI 52040; Merck, Darmstadt, Germany) was added to stain cross-Sects. (2–3 μm), followed by examination and imaging using a Leica DMLA microscope (Leica Microsystems, Wetzlar, Germany).

### Pollen staining viability and germination rate *in vitro*

Golden yellowed anthers derived from blooming flowers of tetraploids and diploids were collected for measurement of pollen viability of staining and germination rate in vitro*,* respectively [68]. Fresh anthers were immersed in the Alexander's staining solution (Solarbio, G3050, Beijing) for 10 min at room temperature, then the pollen was extruded on the slide and observed by microscope. The pollen stained purple-red was regarded as viable, while the green pollen was regarded as non-viable. For germination in vitro, fresh pollen were released onto the germination medium supplemented with 10% sucrose + 0.01% boric acid + 0.03% CaCl_2_ + 1% agar (pH = 6.2). Followed incubation at 25℃ in the dark with humid environment for 20 h, the pollen germination was observed using optical microscope, then the germination rate was calculate according to the counts of the viable pollen with tube longer than diameter of its pollen grain. Each experiment was independently performed in triplicate, and each observation was repeated in three sets of five fields of view.

### Fruit evaluation

Three mature fruits per plant and three plants per genotype (9 fruits) were harvested from the diploids and tetraploids at 210 days after flowering (DAF) for quality determination, respectively. Various characteristics including fruit edible rate, segment number, seed number, titratable acidity (TA), and soluble solids content (SSC) were measured as described by Hijaz et al. [69]. All samples were subjected to three replicates.

### Metabolite extraction and profiling

Metabolites were extracted and quantified following the methods described by Wuhan MetWare Biotechnology Co., Ltd. Nine mature fruits per genotype were harvested at 210 DAF, and each fruit was divided into the peel (including the flavedo and albedo), the juice sac, and the segment membrane, followed by immediate frozen in liquid nitrogen. The samples, including the peel of the diploid (2X-F), the peel of the tetraploid (4X-F), the juice sac of the diploid (2X-P), the juice sac of the tetraploid (4X-P), the segment membrane of the diploid (2X-CW), and the segment membrane of the tetraploid (4X-CW), were dried with a vacuum dryer at −76℃ then grounded into powder for quantitative and qualitative metabolic experiments. Each dry sample (50 mg) was extracted with 70% (v/v) methanol (1.2 mL) for 30 min, and this step was conducted five times. Finally, the supernatants were obtained following 3 min of centrifugation (12,000 rpm) and later passed through 0.22-µm filters [70]. MS/MS (tandem mass spectrometry) and UPLC (ultraperformance liquid chromatography, UPLC) (ExionLC™AD, https://sciex.com.cn/) were used to analyze the filtered extracts. The R software and AB Sciex 1.6.3 were used to evaluate the UPLC-MS/MS results. The Pearson correlation coefficient (PCC) was determined using the R package (base package; Hmisc). and displayed as heat maps. Differential accumulation of metabolites was assessed through hierarchical clustering heatmap analysis (HCA) using the R package, and the normalized metabolite signal intensities (unit variance scaling) were visualized using the color spectrum. We also conducted unsupervised principal component analysis (PCA) using the R software function prcomp (www.r-project.org). Differentially accumulated metabolites (DAMs) were identified based on the absolute log2(fold change) (FC ≥ 2 or FC ≤ 0.5) and VIP (VIP > 1) values extracted from the OPLS-DA results. The KEGG Compound database (http://www.kegg.jp/kegg/compound/) was used to annotate those identified metabolites, whereas the KEGG Pathway database (http://www.kegg.jp/kegg/pathway.html) was used for mapping. The pathways enriched with markedly differentially abundant metabolites were imported for MSEA (metabolite set enrichment analysis), and *p* < 0.05 after hypergeometric testing suggested pathway significance.

### RNA extraction and transcriptome sequencing

The plant samples were immersed in liquid nitrogen immediately after collection. The RNAprep Pure Plant Kit (Tiangen, Beijing, China) was used to extract RNA. A 1% agarose gel was used to analyze the integrity and quality of RNA. Then, 1 µg of RNA was used for constructing the library. We established and sequenced 18 high-quality RNA libraries. High-throughput sequencing was conducted using an Illumina HiSeq6000 platform (Illumina, San Diego, CA, USA). Moreover, transcriptome sequencing analysis was performed in triplicate to obtain high-quality data. The RNA sequencing data were also aligned against those of the *C. grandis* cv. 'Wanbaiyou' v1.0 reference genome (http://citrus.hzau.edu.cn/data/Genome_info/HWB.v1.0/HWB.v1.0.genome.fa) with HISAT2. Then, StringTie was used to determine fragments per kilobase of transcript per million fragments mapped (FPKM). We identified differentially expressed genes (DEGs) with an FDR < 0.05 and a |log2FC|≥ 1. DEGs were analyzed using the R software package. The raw transcriptome data were imported into the NCBI Sequence Read Archive (BioProject: PRJNA1151860).

Correlation analysis was performed and PCCs were calculated using the R software (cor-function). The *p*-value (< 0.05) and correlation coefficient (> 0.85) thresholds were set to filter the results. Heat maps showing correlation clustering and gene expression were constructed using MultiExperiment Viewer and TB tools v2.012, respectively. Correlation networks were constructed using Cytoscape 3.9.1 software.

### Statistical analysis

The data was analyzed using SPSS 26.0 software (SPSS Inc., Chicago, IL, USA). Analysis of variance (ANOVA) and student’s *t*-test were used to detect the difference between genotypes, indicated by * (*P* < 0.05) and ** (*P* < 0.01) as significance, and NS as no significance, respectively.

## Supplementary Information

Additional file 1. All metabolites identified in peels, juice sacs, and segment membranes of diploid and tetraploid

Additional file 2. The total content of each category of metabolites in juice sacs ( **A** ), peels ( **B** ) and segment membranes ( **C** ) of tetraploid and diploid fruits, respectively. An asterisk (*) represents a significance with p-value at the 0.05 level, and two asterisks (***) represent a significance with p-value at the 0.001 level. 4x-p, tetraploid juice sacs; 2x-p, diploid juice sacs; 4x-f, tetraploid peels; 2x-f, diploid peels; 4x-cw, tetraploid segment membranes; 2x-cw, diploid segment membranes.

Additional file 3. Differentially accumulated metabolites (DAMs) identified from comparison between diploid and tetraploid in peels, juice sacs, and segment membranes, respectively

Additional file 4. The statistical number of differentially accumulated metabolites (DAMs) in peel. juice sac, and segment membranes, respectively

Additional file 5. Reads and Q3 value of all samples

Additional file 6. Differentially expressed genes (DEGs) identified from comparison between diploid and tetraploid in peels, juice sacs, and segment membranes, respectively

Additional file 7. Analysis of Gene Ortholog (GO) of DEGs involved in peels ( **A** ), juice sacs ( **B** ), and segment membranes ( **C** ), respectively.

Additional file 8. Analysis of Kyoto Encyclopedia of Genes and Genomes (KEGG) enrichment of DEGs involved in peels ( **A** ), juice sacs ( **B** ), and segment membranes ( **C** ), respectively.

Additional file 9. DEGs specifically up-regulated in tetraploid juice sacs, segment membranes, and peels in K-means analysis

Additional file 10. KEGG enrichment analysis of DEGs involved in subclass 7 in peels.

Additional file 11. KEGG enrichment analysis of DEGs involved in subclass 2 in juice sacs

Additional file 12. KEGG enrichment analysis of up-DEGs of K-means analysis in juice sacs ( **A** ) and segment membranes ( **B** ) of tetraploid fruits, respectively.

Additional file 13. KEGG enrichment analysis of DEGs involved in subclass 6 in segment membranes

Additional file 14. Correlation analysis of top5 up-DAMs and up-DEGs in tetraploid peels

Additional file 15. Correlation analysis of up-DAMs and DEGs in tetraploid juice sacs

Additional file 16. Correlation analysis of up-DAMs and DEGs in tetraploid segment membranes

## Data Availability

The datasets generated and/or analyzed during the current study are available in the NCBI SRA repository, with accession number PRJNA1151860 (https://www.ncbi.nlm.nih.gov/sra/PRJNA1151860). All data generated or analyzed during this study are included in this published article and its supplementary information files.
