# Supplementary figures and images for: Morphological and metabolic changes in Changshan Huyou (Citrus changshan-huyou) following natural tetraploidization
Source: BMC Plant Biol. 2025 Mar 8;25:301. doi: 10.1186/s12870-025-06293-4 (PMC11889857; doi:10.1186/s12870-025-06293-4)

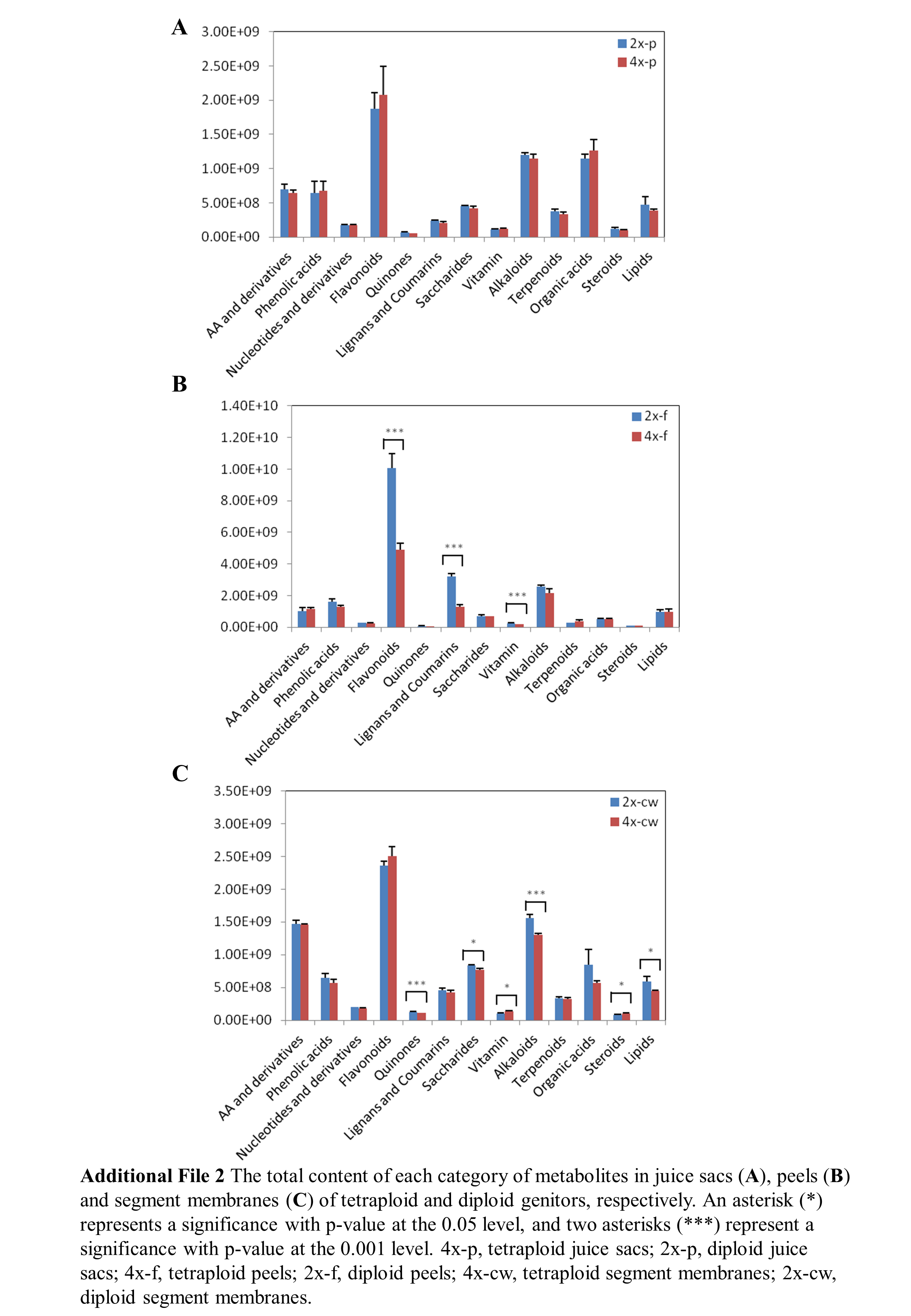

Supplement: Supplementary file 2 — Additional file 2. The total content of each category of metabolites in juice sacs ( A ), peels ( B ) and segment membranes ( C ) of tetraploid and diploid fruits, respectively. An asterisk (*) represents a significance with p-value at the 0.05 level, and two asterisks (***) represent a significance with p-value at the 0.001 level. 4x-p, tetraploid juice sacs; 2x-p, diploid juice sacs; 4x-f, tetraploid peels; 2x-f, diploid peels; 4x-cw, tetraploid segment membranes; 2x-cw, diploid segment membranes. [file 12870_2025_6293_MOESM2_ESM.tif]

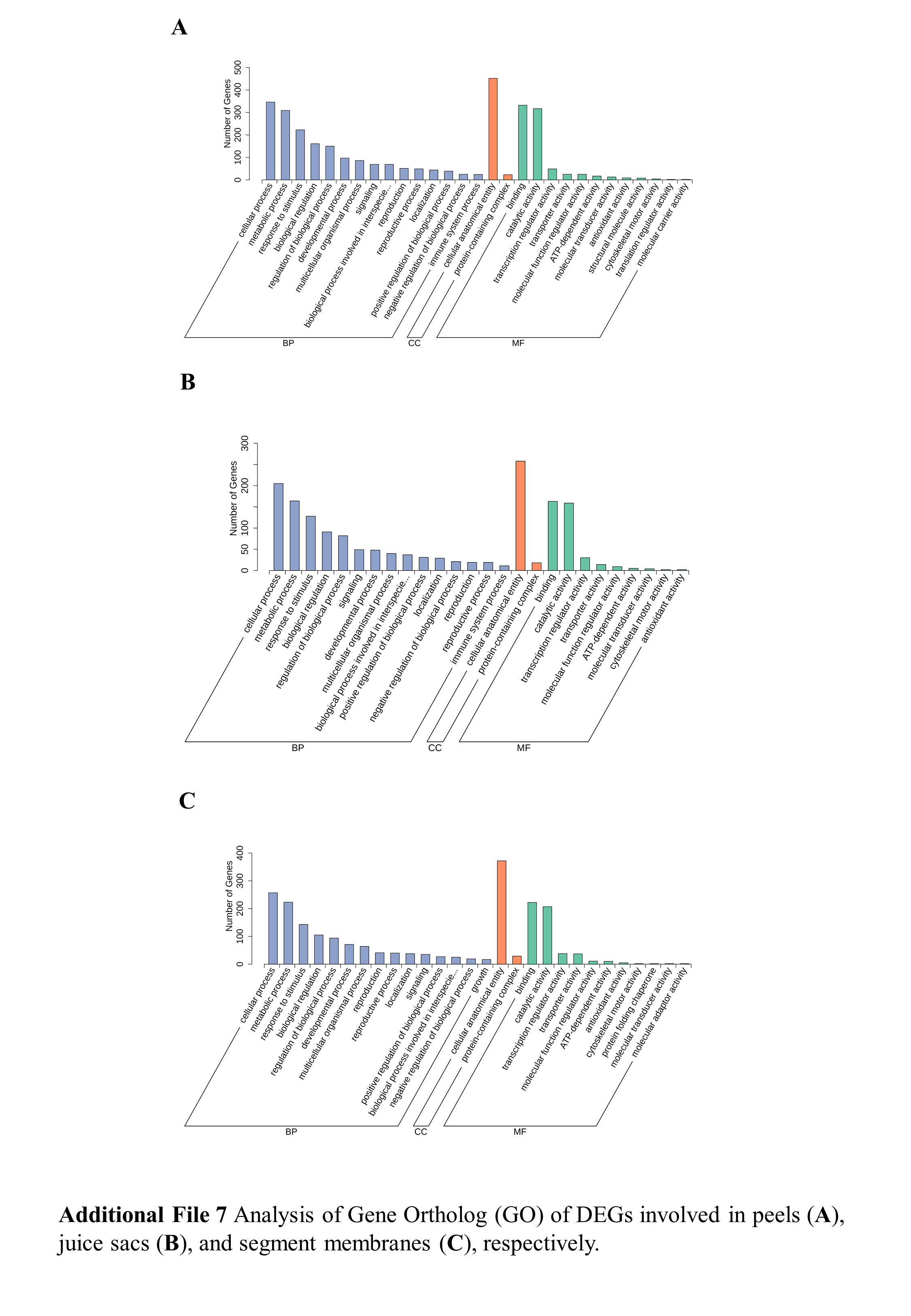

Supplement: Supplementary file 7 — Additional file 7. Analysis of Gene Ortholog (GO) of DEGs involved in peels ( A ), juice sacs ( B ), and segment membranes ( C ), respectively. [file 12870_2025_6293_MOESM7_ESM.tif]

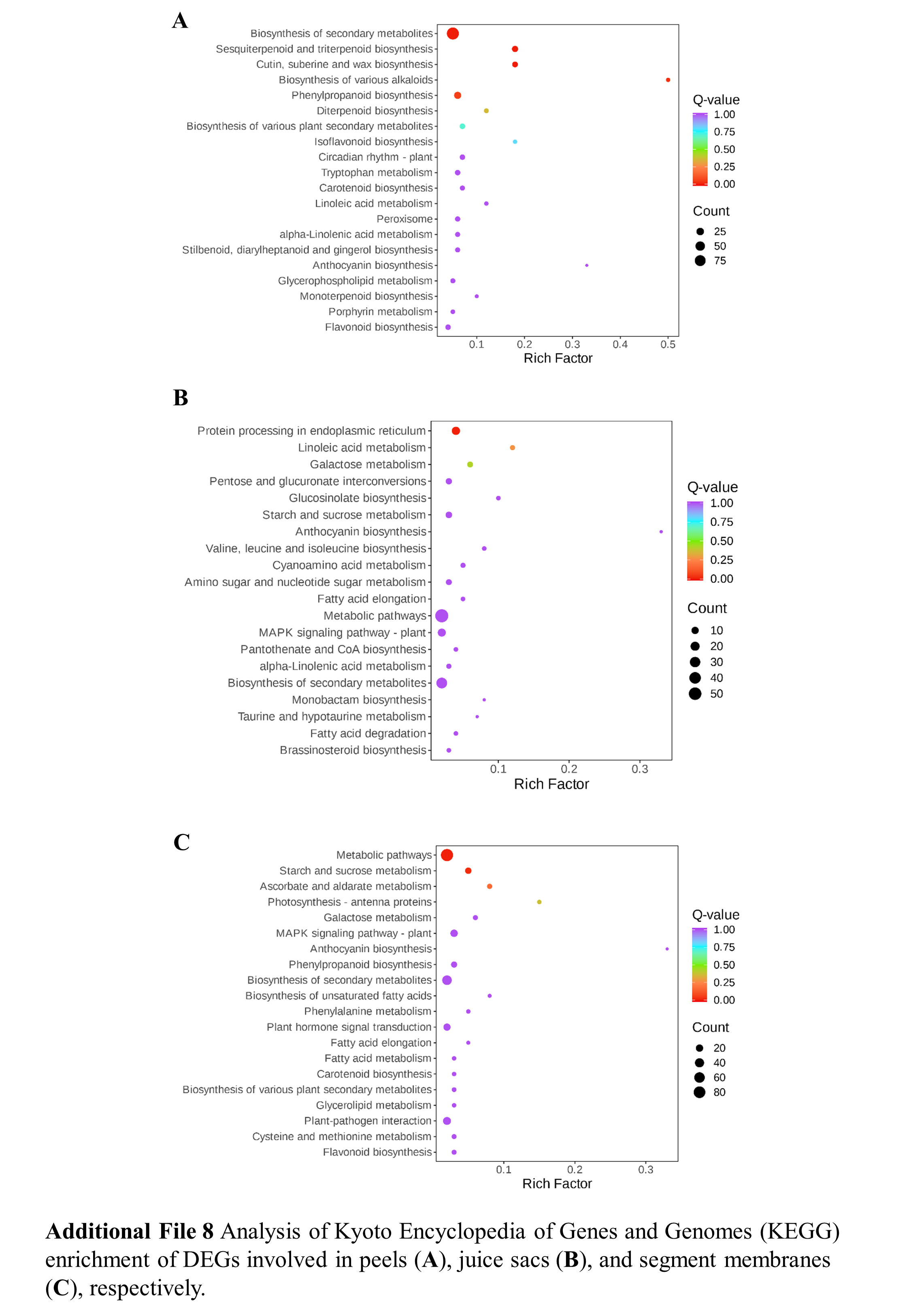

Supplement: Supplementary file 8 — Additional file 8. Analysis of Kyoto Encyclopedia of Genes and Genomes (KEGG) enrichment of DEGs involved in peels ( A ), juice sacs ( B ), and segment membranes ( C ), respectively. [file 12870_2025_6293_MOESM8_ESM.tif]

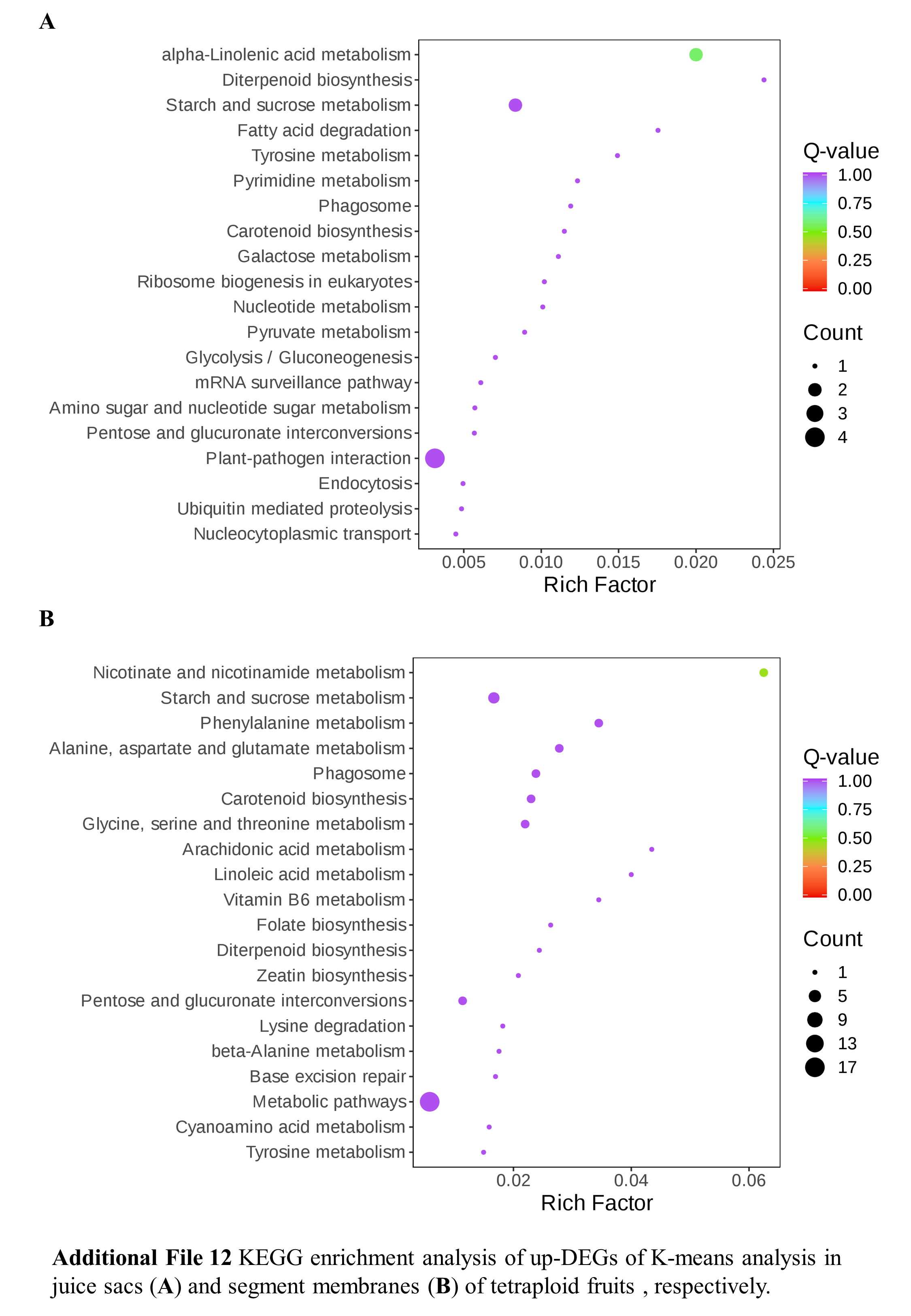

Supplement: Supplementary file 12 — Additional file 12. KEGG enrichment analysis of up-DEGs of K-means analysis in juice sacs ( A ) and segment membranes ( B ) of tetraploid fruits, respectively. [file 12870_2025_6293_MOESM12_ESM.tif]
